# 2,3-Dihydro-1λ^6^,2-benzothia­zine-1,1,4-trione

**DOI:** 10.1107/S1600536812013827

**Published:** 2012-04-04

**Authors:** Farhana Aman, Waseeq Ahmad Siddiqui, Adnan Ashraf, M. Nawaz Tahir

**Affiliations:** a University of Sargodha, Department of Chemistry, Sargodha, Pakistan; b University of Sargodha, Department of Physics, Sargodha, Pakistan

## Abstract

In the title compound, C_8_H_7_NO_3_S, the benzene ring is oriented at a dihedral angle of 69.25 (7)° to the S and O atoms of the sulfonyl group. The heterocyclic ring approximates to an envelope, with the N atom in the flap position. In the crystal, mol­ecules are linked by N—H⋯O_c_ (c = carbon­yl) hydrogen bonds, forming *C*(5) chains along [001]. Two *R*_2_^2^(10) loops arise from pairs of C—H⋯O hydrogen bonds and a weak aromatic π–π stacking inter­action [centroid–centorid separation = 3.8404 (11) Å] also occurs.

## Related literature

For chemical background and related structures, see: Siddiqui *et al.* (2007 ▶, 2008 ▶). For graph-set notation, see: Bernstein *et al.* (1995 ▶). For puckering parameters, see: Cremer & Pople (1975 ▶).
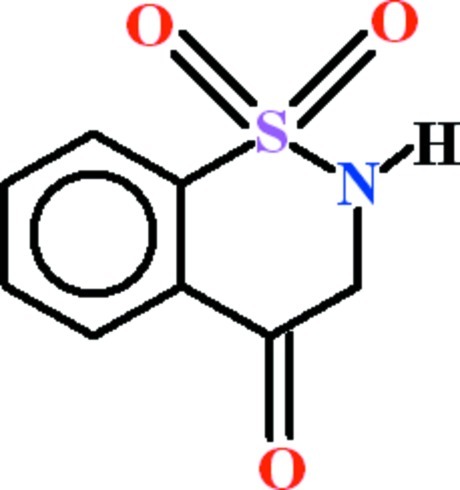


## Experimental

### 

#### Crystal data

C_8_H_7_NO_3_S
*M* *_r_* = 197.21
Monoclinic, 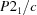

*a* = 8.4950 (4) Å
*b* = 13.7560 (5) Å
*c* = 7.6677 (3) Å
β = 113.214 (1)°
*V* = 823.48 (6) Å^3^
*Z* = 4
Mo *K*α radiation
μ = 0.36 mm^−1^
*T* = 296 K
0.35 × 0.15 × 0.12 mm

#### Data collection

Bruker Kappa APEXII CCD diffractometer
Absorption correction: multi-scan (*SADABS*; Bruker, 2005 ▶) *T*_min_ = 0.915, *T*_max_ = 0.938
7644 measured reflections
2017 independent reflections
1692 reflections with *I* > 2σ(*I*)
*R*_int_ = 0.022

#### Refinement

*R*[*F* ^2^ > 2σ(*F* ^2^)] = 0.036
*wR*(*F* ^2^) = 0.098
*S* = 1.04
2017 reflections
121 parameters
H atoms treated by a mixture of independent and constrained refinement
Δρ_max_ = 0.33 e Å^−3^
Δρ_min_ = −0.37 e Å^−3^

### 

Data collection: *APEX2* (Bruker, 2009 ▶); cell refinement: *SAINT* (Bruker, 2009 ▶); data reduction: *SAINT*; program(s) used to solve structure: *SHELXS97* (Sheldrick, 2008 ▶); program(s) used to refine structure: *SHELXL97* (Sheldrick, 2008 ▶); molecular graphics: *ORTEP-3 for Windows* (Farrugia, 1997 ▶) and *PLATON* (Spek, 2009 ▶); software used to prepare material for publication: *WinGX* (Farrugia, 1999 ▶) and *PLATON*.

## Supplementary Material

Crystal structure: contains datablock(s) global, I. DOI: 10.1107/S1600536812013827/hb6713sup1.cif

Structure factors: contains datablock(s) I. DOI: 10.1107/S1600536812013827/hb6713Isup2.hkl

Supplementary material file. DOI: 10.1107/S1600536812013827/hb6713Isup3.cml

Additional supplementary materials:  crystallographic information; 3D view; checkCIF report

## Figures and Tables

**Table 1:** Hydrogen-bond geometry (Å, °)

*D*—H⋯*A*	*D*—H	H⋯*A*	*D*⋯*A*	*D*—H⋯*A*
N1—H1⋯O3^i^	0.80 (2)	2.34 (2)	3.028 (2)	144 (2)
C2—H2⋯O2^ii^	0.93	2.58	3.443 (2)	154
C8—H8*A*⋯O2^iii^	0.97	2.48	3.273 (2)	139

Symmetry codes: (i) 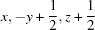; (ii) 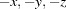; (iii) 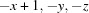.

**Fig. 1.**
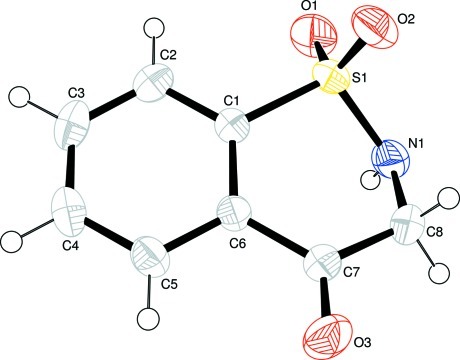
View of the title compound with displacement ellipsoids drawn at the 50% probability level.

**Fig. 2.**
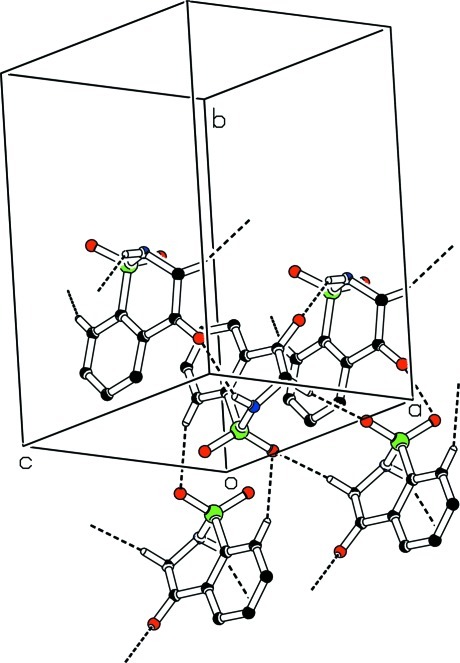
Partial packing diagram for (I) showing C(5) chains extending along [001]. The chains are interlinked with R22(10) rings due to C—H···O bondings. The H-atoms not involved in H-bondings are omitted for clarity.

## supplementary crystallographic information

### Comment

The title compound (I), (Fig. 1) has been synthesized as a pre-cursor. The crystal structures of 2-methyl-2*H*-1,2-benzothiazin-4(3*H*)-one 1,1-dioxide (Siddiqui *et al.*, 2007) has been published which is related to (I).

The dihedral angle between the benzene ring and S1/O1/O2 is 69.25 (7)°. The heterocyclic ring C (C1/C6—C8/N1/S1) is twisted with puckering parameters (Cremer & Pople, 1975) Q = 0.5149 (15) Å, θ = 62.25 (19)° and π = 43.7 (2)°. The molecules are linked in the form of C(5) chains (Bernstein *et al.*, 1995) along the *c*-axis due to H-bondings between amide and carbonyl O-atoms (Table 1, Fig. 2). The neighbouring polyneric chains are interlinked due to C—H···O bonds with two *R*_2_^2^(10) ring motifs (Table 1, Fig. 2), where CH are of benzene and methylene groups and the same O-atom is of sulfonyl group. There exist π–π interaction between the centroids of the benzene rings at a distance of 3.8404 (11)°.

### Experimental

A mixture of (0.5 g, 1.96 mmol) 4-hydroxy-3-carbomethoxy-2*H*-1,2- benzothiazine 1,1-dioxide (Siddiqui *et al.*, 2008) and anhydrous lithium iodide (1.3 g, 9.79 mmol) was subjected to reflux in dimethyl sulphoxide (15 ml) for five h. The dark brown reaction mixture was then poured into crushed ice (100 g). The formed dark yellow precipitates were filtered, washed with cold water (3×25 ml) and dried to get (0.36 g, 1.8 mmol, 92%) of the crude product. Recrystallization of the crude product in ethyl acetate yielded light yellow needles of (I) (m.p. 415–417 K).

IR (KBr) (v_max_, cm^-1^): 3269 (NH), 3078 (Ar. CH), 1683 (C=O), 1577 (NH, def.), 1419 (CH, def.) 1330, 1176 (SO_2_).

### Refinement

The coordinates of H-atom of amide were refined. The H-atoms of aryl and methylene groups were positioned geometrically at C—H = 0.93 and C—H = 0.97 Å, respectively and included in the refinement as riding with *U*_iso_(H) = *xU*_eq_(C, N), where *x* = 1.2 for all H atoms.

### Crystal data

**Table:**

C_8_H_7_NO_3_S	*F*(000) = 408
*M**_r_* = 197.21	*D*_x_ = 1.591 Mg m^−^^3^
Monoclinic, *P*2_1_/*c*	Mo *K*α radiation, λ = 0.71073 Å
Hall symbol: -P 2ybc	Cell parameters from 1692 reflections
*a* = 8.4950 (4) Å	θ = 2.6–28.3°
*b* = 13.7560 (5) Å	µ = 0.36 mm^−^^1^
*c* = 7.6677 (3) Å	*T* = 296 K
β = 113.214 (1)°	Needle, light yellow
*V* = 823.48 (6) Å^3^	0.35 × 0.15 × 0.12 mm
*Z* = 4	

### Data collection

**Table:**

Bruker Kappa APEXII CCD diffractometer	2017 independent reflections
Radiation source: fine-focus sealed tube	1692 reflections with *I* > 2σ(*I*)
Graphite monochromator	*R*_int_ = 0.022
Detector resolution: 7.50 pixels mm^-1^	θ_max_ = 28.3°, θ_min_ = 2.6°
ω scans	*h* = −11→8
Absorption correction: multi-scan (*SADABS*; Bruker, 2005)	*k* = −16→18
*T*_min_ = 0.915, *T*_max_ = 0.938	*l* = −10→10
7644 measured reflections	

### Refinement

**Table:**

Refinement on *F*^2^	Primary atom site location: structure-invariant direct methods
Least-squares matrix: full	Secondary atom site location: difference Fourier map
*R*[*F*^2^ > 2σ(*F*^2^)] = 0.036	Hydrogen site location: inferred from neighbouring sites
*wR*(*F*^2^) = 0.098	H atoms treated by a mixture of independent and constrained refinement
*S* = 1.04	*w* = 1/[σ^2^(*F*_o_^2^) + (0.0455*P*)^2^ + 0.3408*P*] where *P* = (*F*_o_^2^ + 2*F*_c_^2^)/3
2017 reflections	(Δ/σ)_max_ < 0.001
121 parameters	Δρ_max_ = 0.33 e Å^−^^3^
0 restraints	Δρ_min_ = −0.37 e Å^−^^3^

### Special details

**Table:**

Geometry. Bond distances, angles *etc*. have been calculated using the rounded fractional coordinates. All su's are estimated from the variances of the (full) variance-covariance matrix. The cell e.s.d.'s are taken into account in the estimation of distances, angles and torsion angles
Refinement. Refinement of *F*^2^ against ALL reflections. The weighted *R*-factor *wR* and goodness of fit *S* are based on *F*^2^, conventional *R*-factors *R* are based on *F*, with *F* set to zero for negative *F*^2^. The threshold expression of *F*^2^ > σ(*F*^2^) is used only for calculating *R*-factors(gt) *etc*. and is not relevant to the choice of reflections for refinement. *R*-factors based on *F*^2^ are statistically about twice as large as those based on *F*, and *R*- factors based on ALL data will be even larger.

### Fractional atomic coordinates and isotropic or equivalent isotropic displacement parameters (Å^2^)

**Table:**

	*x*	*y*	*z*	*U*_iso_*/*U*_eq_	
S1	0.28018 (5)	0.04479 (3)	0.18815 (6)	0.0352 (2)	
O1	0.21934 (19)	−0.00868 (10)	0.3084 (2)	0.0539 (5)	
O2	0.25497 (18)	0.00747 (10)	0.00536 (19)	0.0499 (5)	
O3	0.55335 (18)	0.29145 (11)	0.1183 (2)	0.0532 (5)	
N1	0.4843 (2)	0.06118 (11)	0.3034 (2)	0.0405 (5)	
C1	0.1985 (2)	0.16427 (11)	0.1625 (2)	0.0297 (4)	
C2	0.0353 (2)	0.18011 (14)	0.1537 (3)	0.0392 (5)	
C3	−0.0234 (2)	0.27484 (15)	0.1446 (3)	0.0447 (6)	
C4	0.0788 (2)	0.35176 (14)	0.1427 (3)	0.0430 (6)	
C5	0.2406 (2)	0.33587 (13)	0.1476 (3)	0.0363 (5)	
C6	0.30357 (19)	0.24184 (11)	0.1576 (2)	0.0285 (4)	
C7	0.4782 (2)	0.22672 (12)	0.1609 (2)	0.0331 (5)	
C8	0.5621 (2)	0.12808 (13)	0.2121 (3)	0.0429 (6)	
H1	0.502 (3)	0.0766 (17)	0.410 (3)	0.0486*	
H2	−0.03448	0.12804	0.15398	0.0471*	
H3	−0.13293	0.28633	0.13970	0.0537*	
H4	0.03854	0.41499	0.13799	0.0516*	
H5	0.30824	0.38842	0.14422	0.0436*	
H8A	0.56037	0.09765	0.09725	0.0514*	
H8B	0.68115	0.13745	0.29619	0.0514*	

### Atomic displacement parameters (Å^2^)

**Table:**

	*U*^11^	*U*^22^	*U*^33^	*U*^12^	*U*^13^	*U*^23^
S1	0.0387 (3)	0.0236 (2)	0.0436 (3)	−0.0023 (2)	0.0165 (2)	−0.0012 (2)
O1	0.0633 (10)	0.0355 (7)	0.0697 (10)	−0.0056 (6)	0.0335 (8)	0.0110 (6)
O2	0.0576 (9)	0.0383 (7)	0.0513 (8)	−0.0023 (6)	0.0188 (7)	−0.0148 (6)
O3	0.0408 (8)	0.0479 (8)	0.0783 (10)	−0.0039 (6)	0.0314 (7)	0.0131 (7)
N1	0.0384 (8)	0.0292 (8)	0.0486 (8)	0.0044 (6)	0.0116 (7)	0.0025 (6)
C1	0.0308 (8)	0.0278 (8)	0.0307 (7)	0.0003 (6)	0.0123 (6)	−0.0008 (6)
C2	0.0305 (9)	0.0446 (10)	0.0442 (9)	−0.0037 (7)	0.0164 (7)	0.0001 (8)
C3	0.0320 (9)	0.0579 (12)	0.0475 (10)	0.0120 (8)	0.0192 (8)	0.0034 (9)
C4	0.0462 (11)	0.0384 (10)	0.0452 (10)	0.0154 (8)	0.0189 (8)	0.0017 (8)
C5	0.0425 (10)	0.0273 (8)	0.0409 (9)	0.0022 (7)	0.0184 (7)	0.0013 (7)
C6	0.0295 (8)	0.0273 (8)	0.0299 (7)	0.0012 (6)	0.0129 (6)	0.0006 (6)
C7	0.0309 (8)	0.0327 (9)	0.0372 (8)	−0.0018 (7)	0.0152 (7)	−0.0016 (7)
C8	0.0321 (9)	0.0371 (10)	0.0625 (12)	0.0031 (7)	0.0219 (8)	−0.0036 (8)

### Geometric parameters (Å, º)

**Table:**

S1—O1	1.4262 (16)	C4—C5	1.378 (3)
S1—O2	1.4273 (14)	C5—C6	1.390 (2)
S1—N1	1.6219 (18)	C6—C7	1.488 (2)
S1—C1	1.7646 (16)	C7—C8	1.511 (2)
O3—C7	1.213 (2)	C2—H2	0.9300
N1—C8	1.462 (3)	C3—H3	0.9300
N1—H1	0.80 (2)	C4—H4	0.9300
C1—C2	1.379 (3)	C5—H5	0.9300
C1—C6	1.401 (2)	C8—H8A	0.9700
C2—C3	1.387 (3)	C8—H8B	0.9700
C3—C4	1.372 (3)		
			
O1—S1—O2	119.80 (9)	C1—C6—C7	122.30 (14)
O1—S1—N1	107.55 (9)	O3—C7—C6	121.31 (16)
O1—S1—C1	109.00 (9)	O3—C7—C8	119.00 (17)
O2—S1—N1	107.42 (9)	C6—C7—C8	119.66 (15)
O2—S1—C1	108.99 (8)	N1—C8—C7	115.70 (16)
N1—S1—C1	102.72 (8)	C1—C2—H2	121.00
S1—N1—C8	114.49 (12)	C3—C2—H2	120.00
C8—N1—H1	112.6 (18)	C2—C3—H3	120.00
S1—N1—H1	108.9 (19)	C4—C3—H3	120.00
C2—C1—C6	121.15 (15)	C3—C4—H4	120.00
S1—C1—C6	119.06 (13)	C5—C4—H4	120.00
S1—C1—C2	119.76 (13)	C4—C5—H5	120.00
C1—C2—C3	119.05 (17)	C6—C5—H5	120.00
C2—C3—C4	120.56 (18)	N1—C8—H8A	108.00
C3—C4—C5	120.39 (18)	N1—C8—H8B	108.00
C4—C5—C6	120.48 (17)	C7—C8—H8A	108.00
C5—C6—C7	119.36 (15)	C7—C8—H8B	108.00
C1—C6—C5	118.34 (16)	H8A—C8—H8B	107.00
			
O1—S1—N1—C8	−170.75 (13)	C2—C1—C6—C7	−178.08 (15)
O2—S1—N1—C8	59.04 (14)	S1—C1—C6—C5	−176.60 (13)
C1—S1—N1—C8	−55.82 (14)	C1—C2—C3—C4	0.6 (3)
O1—S1—C1—C2	−36.22 (16)	C2—C3—C4—C5	0.8 (3)
O2—S1—C1—C2	96.19 (16)	C3—C4—C5—C6	−1.0 (3)
N1—S1—C1—C2	−150.09 (14)	C4—C5—C6—C1	0.0 (3)
O1—S1—C1—C6	141.74 (12)	C4—C5—C6—C7	179.44 (17)
O2—S1—C1—C6	−85.85 (14)	C1—C6—C7—C8	−13.2 (2)
N1—S1—C1—C6	27.87 (14)	C5—C6—C7—O3	−14.6 (2)
S1—N1—C8—C7	53.31 (19)	C1—C6—C7—O3	164.83 (15)
S1—C1—C2—C3	176.30 (15)	C5—C6—C7—C8	167.41 (16)
C6—C1—C2—C3	−1.6 (3)	O3—C7—C8—N1	166.31 (15)
S1—C1—C6—C7	3.99 (19)	C6—C7—C8—N1	−15.6 (2)
C2—C1—C6—C5	1.3 (2)		

### Hydrogen-bond geometry (Å, º)

**Table:**

*D*—H···*A*	*D*—H	H···*A*	*D*···*A*	*D*—H···*A*
N1—H1···O3^i^	0.80 (2)	2.34 (2)	3.028 (2)	144 (2)
C2—H2···O2^ii^	0.93	2.58	3.443 (2)	154
C8—H8*A*···O2^iii^	0.97	2.48	3.273 (2)	139

Symmetry codes: (i) *x*, −*y*+1/2, *z*+1/2; (ii) −*x*, −*y*, −*z*; (iii) −*x*+1, −*y*, −*z*.
